# Neonatal and Delivery Outcomes in Women with Multiple Sclerosis

**DOI:** 10.1002/ana.22483

**Published:** 2011-06-27

**Authors:** Mia L van der Kop, Mark S Pearce, Leanne Dahlgren, Anne Synnes, Dessa Sadovnick, Ana-Luiza Sayao, Helen Tremlett

**Affiliations:** 1 Faculty of Medicine, Division of Neurology, University of British ColumbiaVancouver, British Columbia, Canada; 2 Institute of Health and Society, Newcastle UniversityNewcastle Upon Tyne, UK; 3 Department of Obstetrics and Gynaecology, University of British ColumbiaVancouver, British Columbia, Canada; 4 Department of Pediatrics, University of British ColumbiaVancouver, British Columbia, Canada; 5 Department of Medical Genetics, University of British ColumbiaVancouver, British Columbia, Canada

## Abstract

**Objective:**

To determine (1) whether the risk of adverse neonatal and delivery outcomes differs between mothers with and without multiple sclerosis (MS) and (2) whether risk is differentially associated with clinical factors of MS.

**Methods:**

This retrospective cohort study analyzed data from the British Columbia (BC) MS Clinics' database and the BC Perinatal Database Registry. Comparisons were made between births to women with MS (n = 432) and to a frequency-matched sample of women without MS (n = 2,975) from 1998 to 2009. Outcomes included gestational age, birth weight, assisted vaginal delivery, and Caesarean section. Clinical factors examined included age at MS onset, disease duration, and disability. Multivariate regression models adjusting for confounding factors were built for each outcome.

**Results:**

Babies born to MS mothers did not have a significantly different mean gestational age or birth weight compared to babies born to mothers without MS. MS was not significantly associated with assisted vaginal delivery (odds ratio [OR], 0.78; 95% confidence interval [CI], 0.50–1.16; *p* = 0.20) or Caesarean section (OR, 0.94; 95% CI, 0.69–1.28; *p* = 0.69). There was a slightly elevated risk of adverse delivery outcomes among MS mothers with greater levels of disability, although findings were not statistically significant. Disease duration and age at MS onset were not significantly associated with adverse outcomes.

**Interpretation:**

This study provides reassurance to MS patients that maternal MS is generally not associated with adverse neonatal and delivery outcomes. However, the suggestion of an increased risk with greater disability warrants further investigation; these women may require closer monitoring during pregnancy. ANN NEUROL 2011;

Multiple sclerosis (MS) is a chronic degenerative disease of the central nervous system and the most common cause of nontraumatic neurological disability in young adults in Europe and North America. Around 75% of people with MS are women, and clinical onset most often occurs in early adulthood, just when many are considering starting a family. Studies have shown that between ⅕ and ⅓ of women with MS bear children after disease onset,^1^, ^2^ making the effect of maternal MS on pregnancy outcomes relevant to patients, their family members, and health care professionals.

During the 1990s, research on pregnancy outcomes in MS mothers was generally undertaken as a secondary aspect of investigations of the effect of pregnancy on disability and exacerbations of the disease around the time of pregnancy. Studies examining pregnancy outcomes from this period were small,^3^, ^4^ and findings were for the most part descriptive. More recently, several larger studies examining pregnancy outcomes in MS^5^–^9^ have shown conflicting results. Previous studies also had limited ability to control for many potentially confounding factors and often relied on populating the MS group using only International Classification of Diseases (ICD) codes rather than having clinically defined groups of patients, leading to potential misclassification of study participants or selection of individuals with MS with more severe disease. We linked data from the British Columbia (BC) MS Clinics' database with the BC Perinatal Database Registry (BCPDR) to examine whether maternal MS was associated with adverse neonatal and delivery outcomes and whether risk was associated with age at disease onset, disease duration, or disability.

## Subjects and Methods

### Data Sources

Pregnancy outcome data were drawn from the BCPDR, which captures >99% of births in BC. The registry, administered by Perinatal Services BC, established full provincial coverage in April 2000 and contains information on a wide range of outcomes (all recorded prospectively) and potentially confounding variables. Clinical data came from the BCMS database, established in 1980 (when the first BC MS clinic opened) and estimated to capture 80% of the BC MS population.^10^, ^11^ Clinical factors included disease course at MS onset (relapsing-remitting or primary progressive), age at MS onset (<20 years, 20 to <30 years, ≥30 years), disease duration (<5 years, 5 to <10 years, ≥10 years), and disability. Disability was measured via the Expanded Disability Status Scale (EDSS)^12^ score assigned closest to the time of delivery (±3 years). MS mothers were classified as having a normal neurological examination (EDSS = 0), mild disability (EDSS = 1–3), or moderate to severe disability (EDSS ≥ 3.5).

### Study Participants

All female patients registered at 1 of the 4 MS clinics in BC from 1980 through 2008 were linked at an individual level to births occurring in BC between April 1998 and March 2009. Patients were linked by Personal Health Number, a lifelong unique number assigned under BC's universal health care system. Patients' names and dates of birth were used to confirm the accuracy of linkage. Births to MS patients were frequency-matched by age (±1 year), local health authority, and delivery year to a random sample of births in the general population. For every birth to an MS mother, 4 births in the general population were selected.

Births were included in the MS group if the mother had laboratory-supported or clinically definite MS (Poser or McDonald criteria^13^, ^14^) diagnosed by an MS specialist neurologist. Births to mothers whose disease onset occurred after delivery were excluded. Births to individuals with an ICD-9/10 code for MS in the BCPDR were excluded from the comparison group. Late terminations and multiple births were excluded from both groups.

### Outcomes and Potential Confounders

Primary neonatal outcomes were mean birth weight (grams) and mean gestational age (weeks). Gestational age was based on ultrasound, date of last menstrual period, newborn exam, or a combination of the above, depending upon when the ultrasound was performed (see Appendix A online). Delivery outcomes included assisted vaginal delivery (forceps and vacuum-assisted deliveries excluding forceps delivery for extraction of a breech birth) and Caesarean section. Descriptive outcomes included duration of the second stage of labor and the 5-minute Apgar score.^15^

BCPDR data are captured during antenatal visits and the delivery admission. Potential confounders included demographic factors (maternal age, local health authority, single parent status); alcohol, drug, or tobacco use during pregnancy (considered exposed when a physician/midwife identified use as a risk during the pregnancy); hypertension (gestational hypertension or having a blood pressure reading of ≥140/90mmHg on 2 consecutive occasions during pregnancy); diabetes (abnormal blood glucose during pregnancy, pre-existing or gestational diabetes); anthropometric measurements (body mass index, maternal height); obstetrical history (parity, previous abortion, prior preterm birth or low birth weight baby, previous Caesarean section); infant sex; and delivery factors (induction). Maternal body mass index (BMI), measured prepregnancy or up to the 12th week of gestation, was categorized as underweight (<18.5kg/m^2^), normal (18.5kg/m^2^ to <25.0kg/m^2^), overweight (25.0kg/m^2^ to <30.0kg/m^2^), or obese (≥30kg/m^2^).^16^ Gestational age, birth weight, and duration of labor were considered potential confounders in models in which they were not the outcome under investigation.

### Statistical Analyses

Associations between MS (and related clinical variables) and both gestational age and birth weight were examined using linear regression, with regression coefficients (β) and corresponding 95% confidence intervals (CIs) presented. Associations between MS (and related clinical variables) and binary outcomes (assisted vaginal delivery and Caesarean section) were examined using logistic regression, with results expressed as odds ratios (ORs) and 95% CIs.

**FIGURE:**
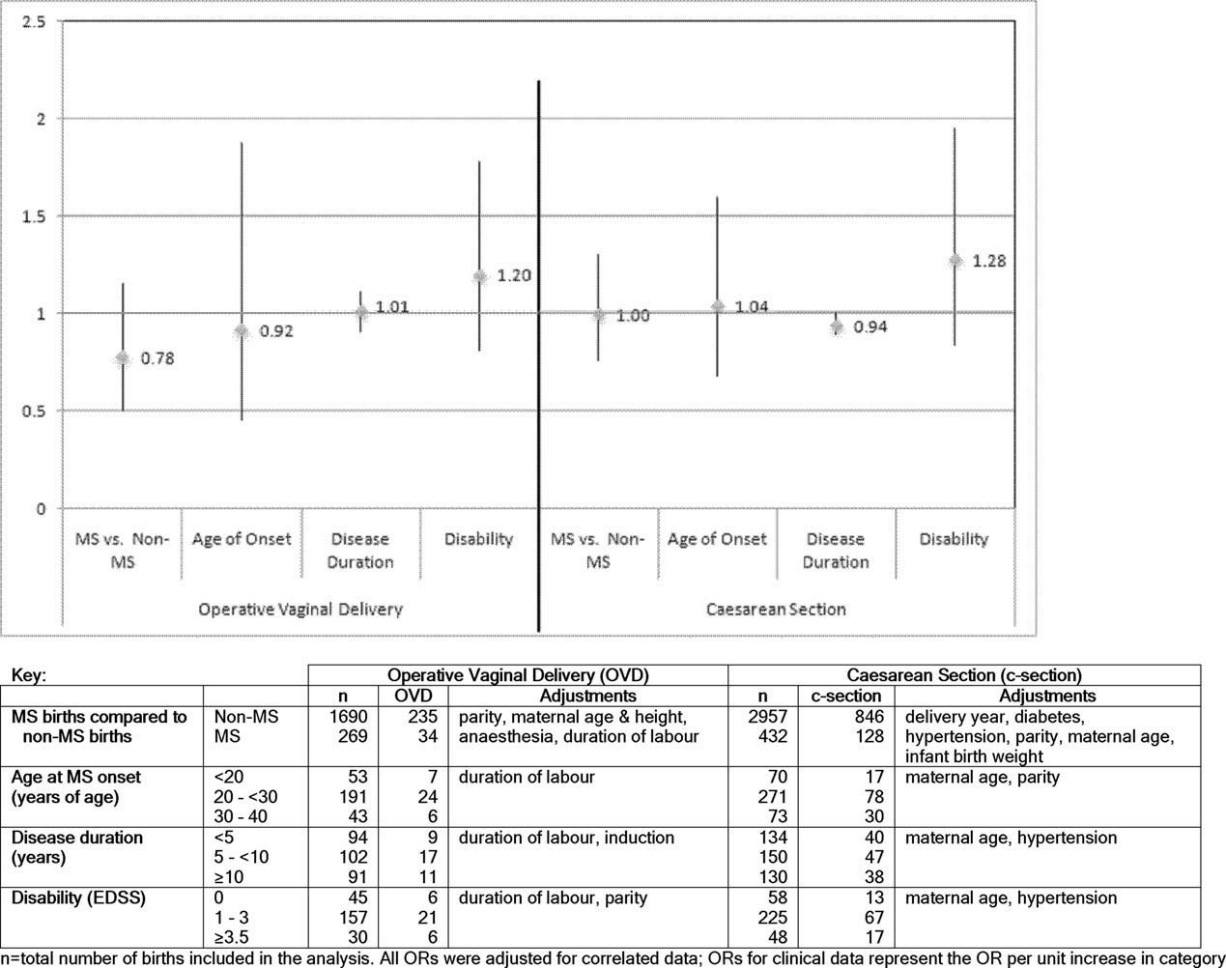
Adjusted odds ratios with 95% confidence intervals for the occurrence of operative vaginal delivery and Caesarean section in multiple sclerosis (MS) mothers compared to non-MS mothers and by clinical factors. EDSS = Expanded Disability Status Scale.

Potential confounding variables, selected using both a priori and empirical methods, were initially examined using stratification and Mantel-Haenszel techniques.^17^ These were then used for adjustment purposes in multivariate models. Statistical significance within regression models was assessed using *F* tests for linear regression and likelihood ratio tests for logistic regression. Interaction between variables and examination of departure from linear trend were also assessed using these tests. To account for the clustered nature of the data, random effects modeling was used. If the quadrature approximation was deemed inaccurate (relative difference in coefficients >0.01), a generalized estimating equations (GEE) approach was employed.^17^

Reported *p* values were 2-sided, and a *p* value of 0.05 was used to determine statistical significance. All analyses were performed using Stata version 11 (Statacorp, College Station, TX).

### Ethics

Ethical approval was obtained from University of British Columbia's Clinical Research Ethics Board.

## Results

### Baseline Characteristics

Linking 7,056 female patients in the BCMS database with the BCPDR resulted in matches for 550 women (762 births) and a frequency-matched sample of 3,048 births in the comparison group. After excluding births to mothers whose MS onset occurred after delivery (n = 76), late terminations (n = 1), nonsingleton infants (n = 101), and births to patients who did not have clinically definite MS (n = 225), the dataset contained 432 births to 321 women with MS and 2,975 births to 2,958 women without MS. Cohort characteristics are shown in Table 1. Anthropometric measurements differed between the groups (see Table 1). A greater proportion of births in the MS group were to women who were nulliparous, primigravid, hypertensive, or smoked during pregnancy. After excluding births that did not fulfill study criteria, deliveries in the comparison group were more likely to occur before 2002. A greater proportion of births in the comparison group were to mothers with diabetes during pregnancy and a history of multiple therapeutic abortions. All other baseline characteristics were similar.

**Table 1:** Characteristics of Births to Women with and without MS in British Columbia, 1998 to 2009

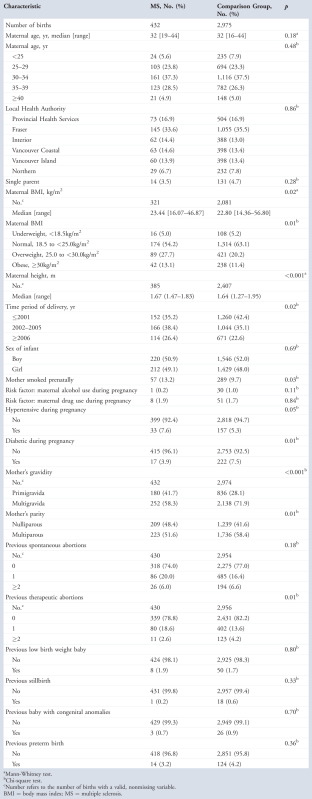

Four births were to women with a primary progressive MS course. The remaining births were to relapsing-onset patients, 18 of which were to women who had reached the secondary progressive phase (Table 2). Not unexpectedly, births in the MS group were to women with a relatively young onset age (median, 24 years; range, 8–39 years). The median disease duration at the time of delivery was 7 years (range, <1–28 years). Of 331 (77%) births to MS patients for whom EDSS scores were available, the majority (68%) were to mothers with mild disability (EDSS = 1–3), 58 were to mothers with a normal neurological examination (EDSS = 0), and 49 were to women with moderate to severe disability (EDSS ≥ 3.5) (range, 0–7.5).

**Table 2:** Clinical Characteristics of the MS Cohort

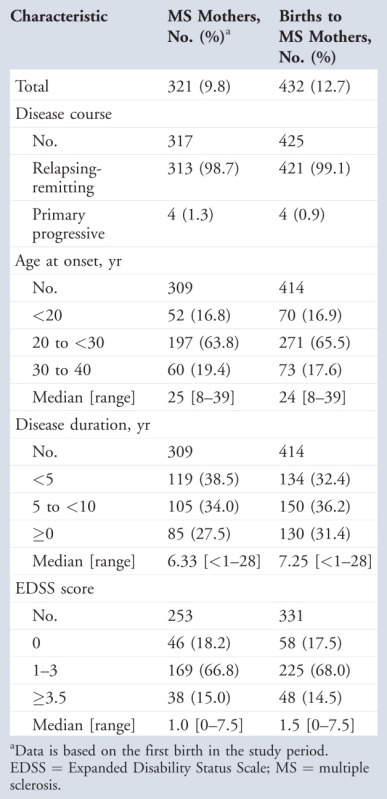

### Delivery Outcomes

Maternal MS was not associated with assisted vaginal delivery (OR, 0.78; 95% CI, 0.50–1.16; *p* = 0.20) or Caesarean section (OR, 0.94; 95% CI, 0.69–1.51; *p* = 0.63) (Fig). The proportion of elective Caesarean sections was similar in both the MS and comparison groups (18.6% vs 16.1%, *p* = 0.61), and the indication for Caesarean delivery did not differ between groups (data not shown). Delivery outcomes were not associated with either an older age at MS onset or longer disease duration. Reliable random effects logistic models could not be fitted for assisted vaginal delivery; therefore, a GEE approach was used to account for correlation between births from the same mother.

**Table 3:** Adjusted and Unadjusted Differences in Mean Gestational Age in Weeks (95% Confidence Interval) between Births in the MS Group and Those in the Comparison Group and by Clinical Factors

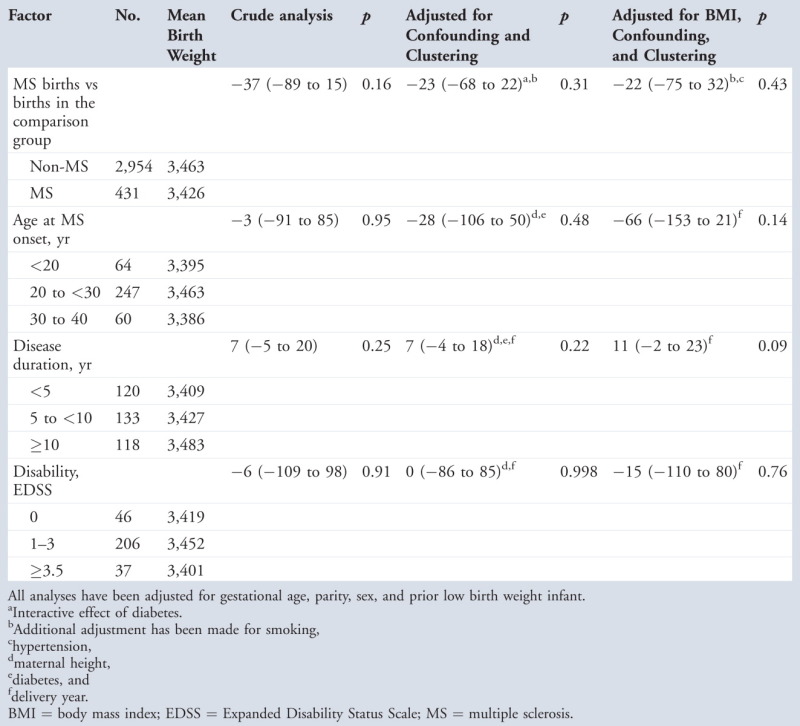

MS mothers with greater disability had slightly higher odds of a Caesarean section compared to women with a normal neurological examination (EDSS = 0) (mild disability: OR, 1.47; 95% CI, 0.71–3.05; *p* = 0.31; moderate/severe disability: OR, 1.64; 95% CI, 0.68–3.95; *p* = 0.27); however, these findings were not statistically significant. A similar effect was found for assisted vaginal delivery, with increased odds found among women with higher EDSS scores around the time of delivery; however, this finding also lacked statistical significance (mild disability: OR, 1.33; 95% CI, 0.45–3.93; *p* = 0.61; moderate/severe disability: OR, 1.65; 95% CI, 0.37–7.31; *p* = 0.51).

### Neonatal Outcomes

The difference in mean birth weight between babies delivered by MS mothers and those in the comparison group was neither clinically nor statistically significant (−22g; 95% CI, −75 to 32g; *p* = 0.43) (Table 3). Since data on maternal BMI were missing for a number of births (31%), a regression model excluding BMI was developed. In this model, diabetic status during pregnancy had an interactive effect; among participants with diabetes during pregnancy, the mean birth weight of babies born to MS mothers was 232g greater than that of babies born in the comparison group (95% CI, 12–452g, *p* = 0.04). Diabetes was not a significant factor once BMI was taken into account. Whether or not BMI was taken into consideration, there were no significant differences in birth weight according to MS clinical factors.

**Table 4:** Adjusted and Unadjusted Differences in Mean Birth Weight in Grams (95% Confidence Interval) between Births in the MS Group and Those in the Comparison Group and by Clinical Factors

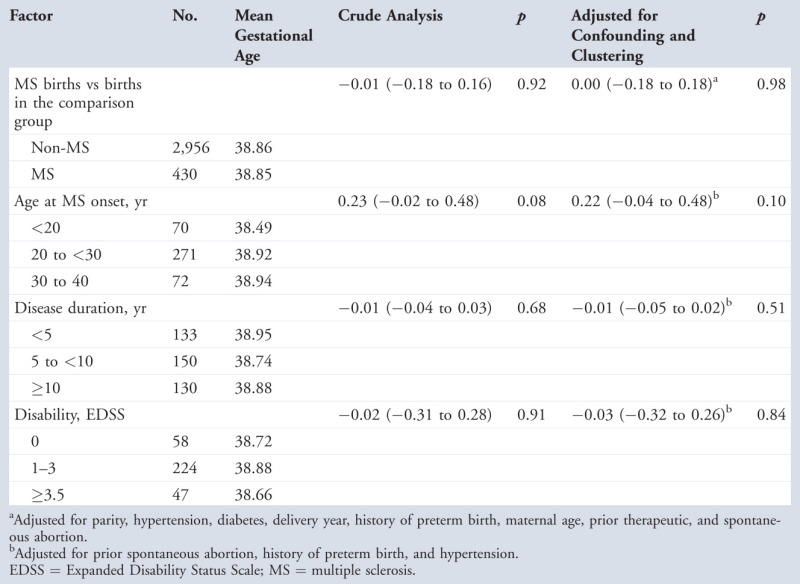

Crude and adjusted analyses revealed no difference in the mean gestational age of babies born to MS mothers compared to babies born in the comparison group (0.00 weeks; 95% CI, −0.18 to 0.18, *p* = 0.98) (see Table 4). Gestational age did not differ according to MS clinical factors. For both gestational age and birth weight, random effects models were used to account for correlated data.

### Descriptive Outcomes

Labor duration data were available for 312 (72%) births to women with MS and 2,191 (74%) births in the comparison group. Because parity is strongly associated with labor duration,^18^, ^19^ analyses were restricted to nulliparous women. There was no significant difference in the median duration of the second stage of labor between women with MS and those in the comparison group (1.35 hours [range, 0.10–6.62] vs 1.28 hours [range, 0.07–26.60], *p* = 0.57).

Duration of the second stage of labor was not associated with age at MS onset (data not shown). The median duration of the second stage of labor increased, however, with disease duration, from 1.08 hours (range, 0.10–4.07 hours) for those with a disease duration of <5 years to 1.51 hours (range, 0.18–6.62 hours) for those with a disease duration of ≥10 years. Compared to women with a normal neurological exam (0.90 hours [range, 0.10–3.40 hours]), women with mild (1.38 hours [range, 0.10–3.40]) or moderate/severe impairment (1.38 hours [range, 0.40–5.63]) had a longer duration of the second stage of labor. Neither of these findings achieved statistical significance.

The median 5-minute Apgar score was the same for babies born to MS mothers and those in the comparison group (median = 9 for both groups; MS range, 5–10; comparison group range, 0–10). The median Apgar score did not change when nonlive births or babies with congenital anomalies were excluded (data not shown). The median 5-minute Apgar score was 9 in each category of the MS-related clinical variables examined: age at MS onset, disease duration, and disability (data not shown).

## Discussion

In this British Columbian cohort, maternal MS was generally not associated with adverse neonatal or delivery outcomes. The mean birth weight and gestational age of babies did not differ between those born to mothers with and without MS. Women with MS were not at a greater risk of adverse delivery outcomes, including Caesarean section and assisted vaginal delivery; the risk of these outcomes was not associated with age at MS onset or disease duration.

Previous studies in MS have not considered the effect of clinical factors on birth outcomes. By linking a clinical database with a pregnancy registry, we were able to examine important clinical factors such as disease duration, onset age, and disability. A limitation of our study was our inability to investigate outcomes among women with higher EDSS scores (eg, ≥6), due to the small numbers of deliveries by women with EDSS scores in this range. Although not statistically significant, our study suggests that there may be an increased risk of adverse delivery outcomes with greater levels of MS disability. Worsening symptoms of MS with higher EDSS scores, including neuromuscular weakness, might be contributing factors. Given both the immediate and long-term health risks associated with assisted delivery,^20^, ^21^ further investigation of the relationship between disability and pregnancy outcomes is needed.

Our main finding of no association between adverse neonatal or delivery outcomes and maternal MS has been reported in some other studies, but not all. In Washington (a US state sharing a geographical border with BC), no adverse associations were found when examining the risk of Caesarean section, forceps-assisted delivery, and length of gestation in women with MS compared to women without MS.^6^ In contrast, Dahl et al had found significant associations for these outcomes in addition to an increased risk of labor induction in a large Norwegian cohort.^5^ Other investigators have found increased risks associated with MS, including Caesarean section,^8^, ^9^ vacuum extraction,^7^ babies who were small for gestational age,^8^ and preterm birth.^8^

A strength of our study was the ability to account for the clustered nature of the data arising from sequential births to the same mother during the study period. Although this adjustment only had a minor effect on our findings, inability to account for correlation between births to the same mother^5^, ^6^, ^9^ can result in an overestimation of the risk associated with MS. Differences between the results in this study and previous investigations may stem from our ability to control for several important confounding factors that many previous investigations could not,^9^ including gestational hypertension, diabetes, obstetrical history, and BMI. BMI was associated with MS in our cohort, with a greater proportion of MS mothers classified as overweight or obese compared to mothers in the general population. A high prevalence of overweight and obesity has been found in individuals with MS,^22^ possibly related to physical disability restricting physical activity.^23^ Overweight and obese mothers have been found to be at an increased risk of adverse birth outcomes, including Caesarean section,^24^–^26^ macrosomic infants,^26^–^28^ and assisted vaginal delivery.^29^ In our study, BMI confounded the association of MS and several outcomes, including having an influence on the association between MS and birth weight. BMI data were not available for all mothers (31%). Although we cannot exclude the possibility of bias, we did find that the demographic characteristics of participants who were missing data were similar to those of participants with data, and that the clinical characteristics of MS patients missing BMI data were similar to those of subjects with BMI data. We acknowledge that comparing MS mothers to non-MS mothers on numerous characteristics increased our probability of a type I error. Nevertheless, because of the risks associated with high BMI and its role as a confounding factor in this study, the association between BMI and MS should be considered in further investigations of pregnancy in MS.

The BCMS database captures data for approximately 80% of MS patients in BC.^10^, ^11^ Why the remaining patients do not attend is unclear. Barriers could include issues with physical access to the clinic sites or very mild disease; however, a systematic difference in birth outcomes of patients who attend BCMS clinics versus those who do not is not suspected. Potential bias in the selection of the comparison group was minimized through the random selection of births to women without MS from the BCPDR (capturing >99% of births in BC). Data on demographic characteristics and the prevalence of outcomes in the sample of mothers without MS were consistent with provincial birth data,^30^ confirming the validity of the comparison group. Potential limitations of our study included our inability to adjust for potential confounders such as ethnicity and socioeconomic status.

Having an MS specialist neurologist-confirmed diagnosis of MS for all patients is a considerable advantage over studies that relied solely on ICD codes in administrative databases to populate the MS group.^5^, ^6^, ^8^, ^9^ For instance, to improve specificity over studies that identified women with MS by the presence of 1 ICD code, a recent Taiwanese study only included births in the MS group if mothers had multiple diagnostic codes for MS within a limited period of time prior to delivery (eg, 3 MS ICD codes during the 2 years before delivery).^8^ This might have resulted in only those women with more active or severe disease being considered, which may in part explain findings that MS was associated with several adverse pregnancy and birth outcomes. Similarly, in a large American study that classified women as having MS if an ICD code for MS was present on hospital discharge abstracts, an elevated risk of Caesarean section associated with MS was found.^9^ Conflicting results between this study and ours may in part be explained by the way MS was ascertained, with hospital abstracts likely to identify a different population of MS mothers than a cohort identified from a population-based clinical database. Additional strengths of our study include the use of a prospectively collated population-based pregnancy registry. The BCPDR can be considered objective and unbiased, because data were recorded around the time of occurrence and independently of the current study question. Its validity has been independently examined^31^ and its reliability confirmed through extensive data checking. A further strength of this study was access to clinical data, which resulted in the first ever investigation of pregnancy and birth outcomes in relation to age at MS onset, disease duration, and disability.

### Conclusions

Our study is reassuring to women with MS who are considering starting a family as it shows that, in general, MS was not associated with adverse pregnancy or birth outcomes. However, MS mothers were more often overweight or obese. Because high BMI is associated with adverse pregnancy and birth outcomes, women with MS can be supported to optimize their weight when planning a pregnancy. This also highlights the importance of considering BMI in future investigations of pregnancy-related outcomes in MS. In addition, the possibility of an increased risk of assisted vaginal and Caesarean delivery with greater MS disability warrants further investigation.
