# Supplementary material for: Neonatal and Delivery Outcomes in Women with Multiple Sclerosis
Source: Ann Neurol. 2011 Jun 27;70(1):41–50. doi: 10.1002/ana.22483 (PMC3625744; doi:10.1002/ana.22483)
Supplement: Supplementary file 1 [file ana0070-0041-SD1.doc]

## Appendix A: Calculation of gestational age

Perinatal Services BC calculates final gestational age (in completed weeks) using the following method:

- 1. The gestational age from the mother’s last menstrual period (LMP) if calculated gestational age by LMP date and calculated gestational age by first ultrasound date differed by less than 7 days.
  2. If the difference between the gestational age by last menstrual period and the gestational age by early ultrasound was greater than 1 week but less than 2 weeks then:
- Gestational age by early ultrasound was used if the ultrasound was done at less than 12 weeks gestation.
- Gestational age by last menstrual period was used if the ultrasound was done from 12 to 19 weeks.
  1. If the difference between the gestational age by last menstrual period and gestational age by early ultrasound was greater than or equal to 2 weeks, then gestational age by early ultrasound was used.
  2. If no gestational age from last menstrual period was recorded, the gestational age from early ultrasound was used.
  3. If no gestational age from LMP or gestational age from early ultrasound was recorded, the gestational age from the newborn exam was used.

If no gestational age from LMP, or gestational age by early ultrasound, or gestational age from the newborn exam was recorded, the gestational age from the maternal chart documentation was used.

Source: BCPDR Data Dictionary. Vancouver: Perinatal Services BC; 2009.
